# Resorufin analogs preferentially bind cerebrovascular amyloid: potential use as imaging ligands for cerebral amyloid angiopathy

**DOI:** 10.1186/1750-1326-6-86

**Published:** 2011-12-22

**Authors:** Byung Hee Han, Meng-liang Zhou, Ananth K Vellimana, Eric Milner, David H Kim, Jacob K Greenberg, Wenhua Chu, Robert H Mach, Gregory J Zipfel

**Affiliations:** 1 Department of Neurological Surgery, Washington University School of Medicine, St. Louis, MO 63110, USA; 2 Hope Center for Neurological Disorders, Washington University School of Medicine, St. Louis, MO 63110, USA; 3 Program in Neuroscience, Washington University Division of Biology and Biomedical Sciences, St. Louis, MO 63110, USA; 4 Division of Radiological Sciences, Washington University School of Medicine, St. Louis, MO 63110, USA; 5 Department of Neurology, Washington University School of Medicine, St. Louis, MO 63110, USA

**Keywords:** Cerebral amyloid angiopathy, Alzheimer's disease, dementia, diagnosis, amyloid beta, positron emission tomography, amyloid imaging, tracer, resorufin, phenoxazines

## Abstract

**Background:**

Cerebral amyloid angiopathy (CAA) is characterized by deposition of fibrillar amyloid β (Aβ) within cerebral vessels. It is commonly seen in the elderly and almost universally present in patients with Alzheimer's Disease (AD). In both patient populations, CAA is an independent risk factor for lobar hemorrhage, ischemic stroke, and dementia. To date, definitive diagnosis of CAA requires obtaining pathological tissues via brain biopsy (which is rarely clinically indicated) or at autopsy. Though amyloid tracers labeled with positron-emitting radioligands such as [^11^C]PIB have shown promise for non-invasive amyloid imaging in AD patients, to date they have been unable to clarify whether the observed amyloid load represents neuritic plaques versus CAA due in large part to the low resolution of PET imaging and the almost equal affinity of these tracers for both vascular and parenchymal amyloid. Therefore, the development of a precise and specific non-invasive technique for diagnosing CAA in live patients is desired.

**Results:**

We found that the phenoxazine derivative resorufin preferentially bound cerebrovascular amyloid deposits over neuritic plaques in the aged Tg2576 transgenic mouse model of AD/CAA, whereas the congophilic amyloid dye methoxy-X34 bound both cerebrovascular amyloid deposits and neuritic plaques. Similarly, resorufin-positive staining was predominantly noted in fibrillar Aβ-laden vessels in postmortem AD brain tissues. Fluorescent labeling and multi-photon microscopy further revealed that both resorufin- and methoxy-X34-positive staining is colocalized to the vascular smooth muscle (VSMC) layer of vessel segments that have severe disruption of VSMC arrangement, a characteristic feature of CAA. Resorufin also selectively visualized vascular amyloid deposits in live Tg2576 mice when administered topically, though not systemically. Resorufin derivatives with chemical modification at the 7-OH position of resorufin also displayed a marked preferential binding affinity for CAA, but with enhanced lipid solubility that indicates their use as a non-invasive imaging tracer for CAA is feasible.

**Conclusions:**

To our knowledge, resorufin analogs are the fist class of amyloid dye that can discriminate between cerebrovascular and neuritic forms of amyloid. This unique binding selectivity suggests that this class of dye has great potential as a CAA-specific amyloid tracer that will permit non-invasive detection and quantification of CAA in live patients.

## Background

Cerebral amyloid angiopathy (CAA) is characterized by amyloid deposition within the walls of leptomeningeal and cortical arterioles. Among the several types of amyloid proteins causing CAA, amyloid β (Aβ) is by far the most common. Aβ comprises several species of 39-43-residue peptides (including Aβ_1-40 _and Aβ_1-42_) that are produced from amyloid precursor protein (APP) via sequential proteolytic cleavage by β- and γ-secretases [1-3]. Soluble Aβ monomers are produced throughout life; in certain individuals, these aggregate to form insoluble amyloid fibrils. This pathological form of Aβ is the major constituent of CAA. It is also the primary component of neuritic plaques - one of the pathological hallmarks of Alzheimer's disease (AD). The composition and pathogenesis of vascular vs. parenchymal amyloid deposits, however, have important differences. For example, while Aβ_1-42 _is thought to be an important seed for the formation of both parenchymal plaques and CAA formation [4,5], higher Aβ_1-40 _levels and increased Aβ_1-40_/Aβ_1-42 _ratios favor formation of CAA over parenchymal plaques in mouse models of AD [6-9].

CAA is primarily a disease of the elderly, with about one-third of individuals aged 60 years or older demonstrating CAA upon postmortem histopathological examination. The incidence of CAA is even higher in patients with AD since these two conditions share common risk factors. Indeed, up to 90% of AD patients have histological evidence of amyloid deposits within cerebral vessels [10,11]. Clinically, CAA is a well-recognized cause of "lobar" hemorrhage in the elderly [12,13]. Several population-based autopsy studies indicate that CAA is also an independent risk factor for ischemic stroke and dementia [14-18]. To further define the relationship between CAA and its neurological consequences, and to effectively examine novel therapeutics directed against CAA, definitive identification of CAA prior to patient death is critical. Yet, to date, definitive diagnosis of CAA is possible only by direct examination of pathological tissue. Short of obtaining such tissue via brain biopsy, only "possible" or "probable" diagnosis of CAA is achievable through use of the Boston Criteria, which utilize MRI to detect lobar microhemorrhage as an indirect indicator of CAA[19]. This indirect diagnostic technique, however, is limited by its inability to quantify CAA severity and its reliance on cerebral hemorrhage as a surrogate marker for CAA[19]. Development of a non-invasive method for selectively and accurately diagnosing and quantifying CAA would therefore be a major breakthrough for this disease.

Investigation into amyloid-imaging ligands for the diagnosis of AD and the evaluation of anti-amyloid therapy started more than 10 years ago [20-24]. Fibrillar amyloid-binding dyes such as Congo red, chrysamine G, and thioflavins were investigated as ligands for positron emission tomography (PET) and single photon emission computed tomography (SPECT) imaging of amyloid deposits in AD patients. Utilizing radiolabeled forms of these molecules, however, was not clinically feasible due to their relative inability to cross the blood-brain barrier (BBB) and their low binding affinities for Aβ aggregates. Since the mid-1990s, many groups have attempted to develop CNS-accessible amyloid ligands derived from those molecules. To date, at least two amyloid tracers - [^11^C]PIB ([^11^C]6-OH-BTA-1) and [^18^F]florbetapir ([[^18^F]AV-45) - have been well characterized. Derived from thioflavin-T and styrylpyridine, respectively, both display favorable amyloid binding profiles, suggesting their great potential as a non-invasive method for early detection of AD and evaluation of anti-amyloid therapies in AD patients [25-32]. However, neither dye is appropriate for the specific diagnosis and quantification of CAA due to their lack of selectivity for parenchymal versus cerebrovascular Aβ deposits as well as the low resolution of PET imaging.

During our laboratory's exploration into the effects of CAA deposits on neurovascular architecture and function in aged APP transgenic mice, we serendipitously observed that the fluorescent dye resorufin (7-hydroxy-3H-phenoxazin-3-one) appeared to selectively bind cerebral arterioles bearing congophilic fibrillar amyloid. In this study, we sought to further characterize this unique amyloid binding property of resorufin, and also explore the feasibility of utilizing resorufin and/or its derivatives for CAA-specific amyloid imaging.

## Results

### *In situ *evidence that resorufin preferentially binds CAA in aged Tg2576 mouse brains

**Figure 1:**
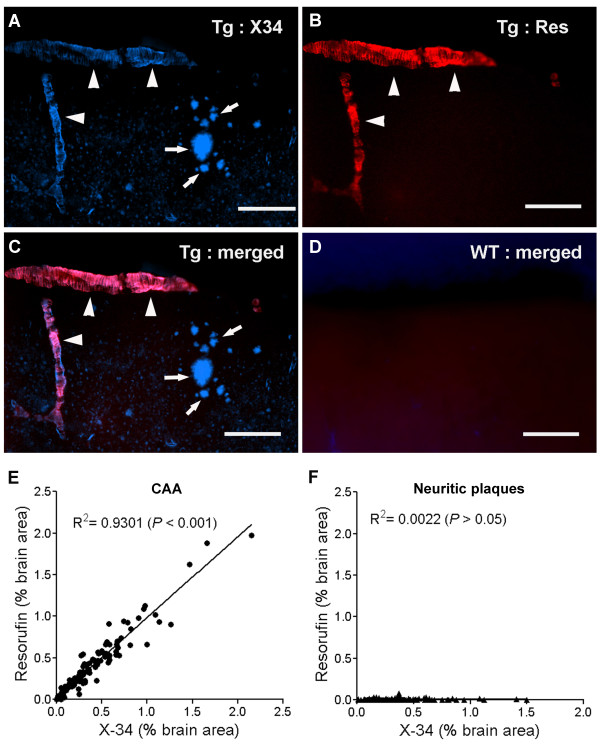
**Resorufin preferentially binds CAA in aged Tg2576 brain sections**. Paraformaldehyde-fixed brain sections prepared from 16-month-old Tg2576 transgenic mice (Tg) or littermate wild-type mice were co-stained with 1 μM resorufin (Res) and 2 μM methoxy-X34 (X34), followed by fluorescent microscopy (*N *= 6-8). Representative images of X34 and resorufin staining in Tg2576 mice (A-C) and wild-type mice (D) are shown. Resorufin selectively binds CAA-laden arterioles (arrowheads) but not senile plaques (arrows), whereas the Congo red derivative methoxy-X34 labels both. Both resorufin and methoxy-X34 staining was absent in age-matched WT mice. Scale bars in A-D: 100 μm. CAA loads (E) and neuritic plaque loads (F) as determined by methoxy-X34 (X34) staining vs. resorufin staining in the motor cortex of Tg2576 mice were plotted.

Aged Tg2576 mice develop congophilic Aβ aggregates within neuritic plaques throughout the cortex and hippocampus, as well as within cortical leptomeningeal and penetrating arteries [6,33-35]. To determine whether resorufin detects Aβ aggregates *in situ*, fixed brain tissues prepared from aged (12-16 mo) Tg2576 mice were co-stained with the Congo red derivative methoxy-X34 and resorufin, followed by fluorescent microscopy (Figure 1). Consistent with previous reports [33,35], we found that methoxy-X34 visualized both cerebrovascular Aβ deposits and neuritic plaques in aged Tg2576 mice (Figure 1A). The fluorescent dye resorufin (E_x_: 573 nm; E_m_: 590 nm), however, was found to strongly bind CAA-laden cerebral arterioles but not parenchymal neuritic plaques (Figure 1B). Moreover, resorufin-positive staining was exactly colocalized to methoxy-X34-positive staining in CAA-laden vessels (Figure 1C and 1E), indicating that resorufin directly interacted with cerebrovascular Aβ aggregates. In contrast, resorufin did not bind methoxy-X34-positive neuritic plaques (Figure 1C and 1F). Both resorufin and methoxy-X34 reactivity was absent in age-matched wild-type mice (Figure 1D) and in young (6-month-old) Tg2576 mice that do not have fibrillar amyloid deposits (data not shown). These data clearly and directly indicate that resorufin preferentially binds cerebrovascular Aβ aggregates over neuritic plaques in brain sections of aged Tg2576 mice.

### Resorufin-positive staining localizes to areas of disrupted vascular integrity in CAA-affected vessels in aged Tg2576 mouse brains

**Figure 2:**
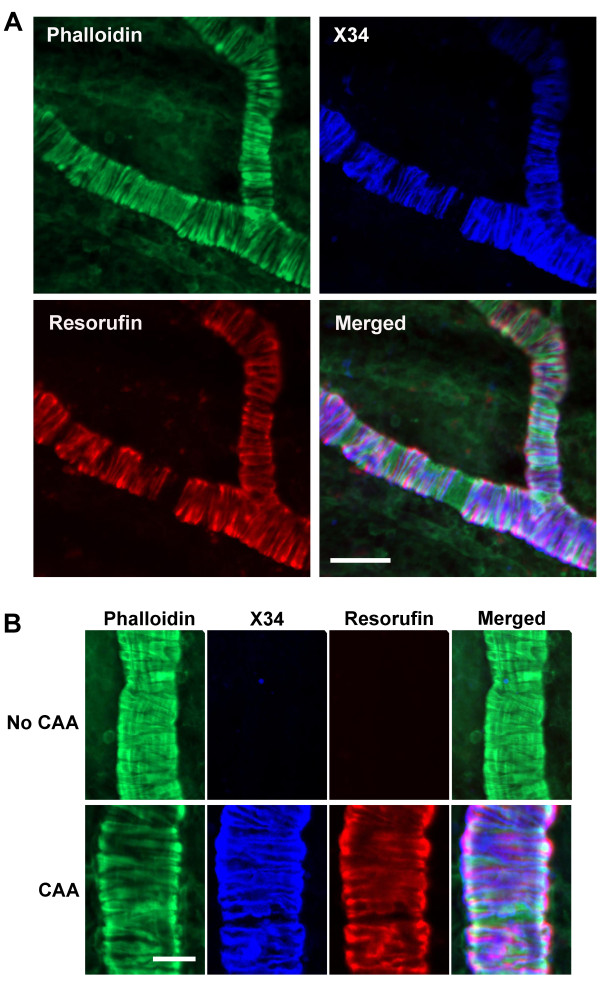
**Multi-photon imaging reveals colocalization of resorufin and methoxy-X34 in CAA-affected vessels**. Fixed whole brains prepared from aged Tg2576 mice were labeled with a vascular smooth muscle cell (VSMC) marker phalloidin-Alexa 488 (green), and amyloid binding dyes methoxy-X34 (blue) and resorufin (red), followed by imaging by multi-photon microscopy (*n *= 7). **A**. Representative fluorescent images in the same field demonstrate co-localization of resorufin and X34 staining in the leptomeningeal vessels of aged Tg2576 mice. **B**. Higher magnification images show that both resorufin and methoxy-X34 are co-localized to the VSMC layer of the vessel segment having severe disruption of VSMC arrangement - a characteristic feature of CAA. In contrast, both methoxy-X34 and resorufin staining is absent in the vessel segment with no CAA pathology. Scale bars, in A: 50 μm; in B: 25 μm.

To explore whether resorufin-positive amyloid deposits influence vascular smooth muscle cell (VSMC) architecture, whole brains from aged Tg2576 transgenic mice and wild-type littermate controls were fixed and subsequently stained with resorufin and the VSMC marker phalloidin-Alexa 488. In line with our previous findings [35], multi-photon microscopy demonstrated that VSMCs were arranged closely in parallel in control mice as well as CAA-free pial arterioles in Tg2576; such vessels (and vessel segments) did not stain with resorufin (Figure 2). However, resorufin- and methoxy-X34-positive Aβ deposits in cerebral vessels were frequently noted in cerebral vessels of aged Tg2576 mice. In these vessels, VSMC arrangement was found to be substantially disrupted - a characteristic feature of CAA.

### *In situ *evidence that resorufin preferentially binds CAA in human AD brains

**Figure 3:**
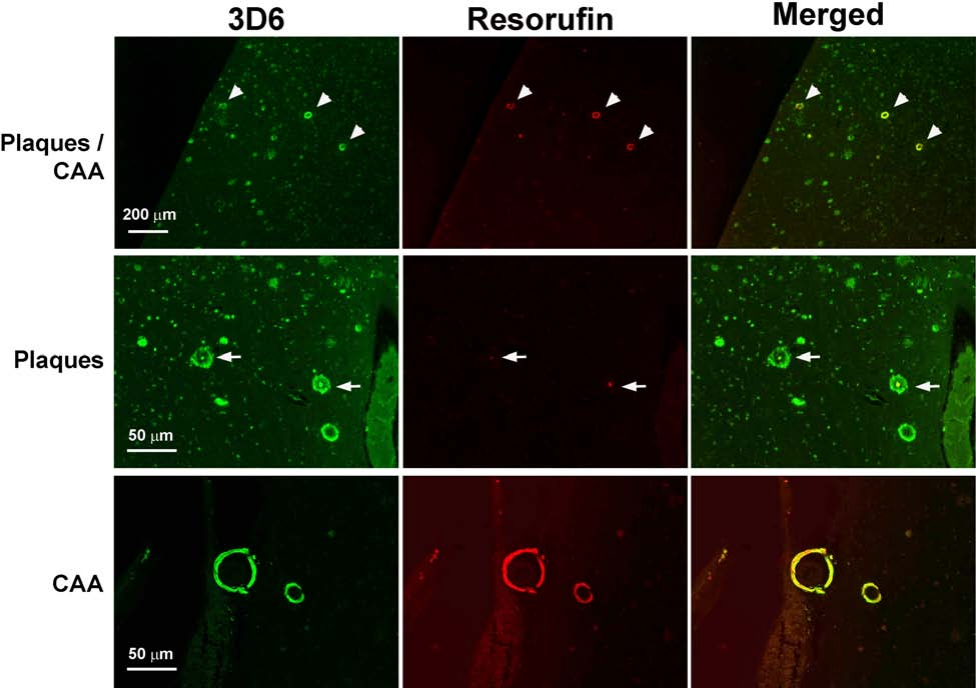
**Preferential binding of resorufin to CAA vs. neuritic plaques in human AD brains**. Paraffin-embedded cortical brain sections of human AD patient were subjected to immunofluorescent labeling with anti-Aβ antibody (3D6, green) followed by staining with resorufin (red). Resorufin selectively labeled 3D6-immunoreactive amyloid deposits in arterioles (arrowheads) but only rarely neuritic plaques (arrows).

We next examined whether resorufin preferentially binds CAA deposits in human AD brains by immunofluorescent labeling. Paraffin-embedded cortical sections were incubated with the anti-Aβ antibody 3D6 followed by staining with resorufin (Figure 3). Consistent with our results in Tg2576 mice (Figure 1), marked resorufin-positive staining was noted in the 3D6-positive cerebral arterioles of human AD brains (arrowheads in Figure 3). In contrast, resorufin did not colocalize to 3D6-positive neuritic plaques. Interestingly, resorufin-positive staining was occasionally present in the core of congophilic neuritic plaques (arrows in Figure 3). We also observed that tissues at more advanced stages of CAA pathology (i.e., those with greater numbers of CAA-laden vessels) demonstrated more intense resorufin-positive staining of vessels (data not shown).

### *In vivo *live imaging of CAA amyloid deposits in aged Tg2576 mice

**Figure 4:**
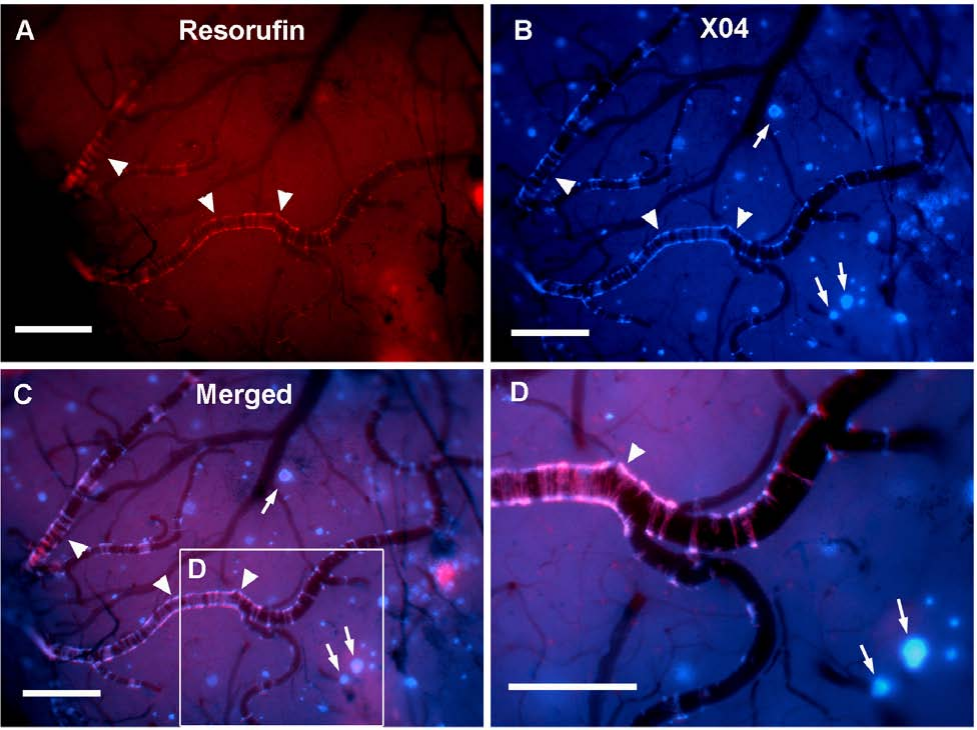
***In vivo *live imaging of CAA amyloid deposits through closed cranial window**. Closed cranial windows were prepared on the right parietal bone of 16 month-old Tg2576 mice and the congophilic amyloid binding dye methoxy-X04 (X04) was administered (6 mg/kg i.p.). On the next day, 2 μM resorufin (dissolved in artificial CSF) was superfused over the brain through a closed cranial window for 5 min. After washing with artificial CSF for 10 min, live fluorescent images of resorufin (red) and X04 (blue) were taken (*N *= 6). Resorufin selectively labels amyloid deposits in arterioles (arrowheads) but not neuritic plaques (arrows), whereas methoxy-X04 staining is present in both. Scale bars: 100 μm.

We observed that intravenous administration of resorufin (up to 50 mg/kg) in aged Tg2576 mice (15 mo, i.e., at an age when CAA is highly prevalent) failed to visualize CAA deposits. Instead, the highly fluorescent resorufin remained within the lumen of cerebral blood vessels (data not shown), indicating that resorufin failed to cross the BBB, probably due to its low lipophilicity (logP_oct_). We therefore examined whether resorufin was able to selectively visualize CAA deposits in live Tg2576 mice utilizing a closed cranial window preparation with real time imaging-fluorescent microscopy[35]. Topical application of resorufin (2 μM) onto the brain surface of aged Tg2576 mice through the cranial window resulted in intense fluorescent labeling within the walls of the leptomeningeal arteries (arrowheads in Figure 4A), but not in neuritic plaques (arrows in Figure 4A). In contrast, topical application of methoxy-X04 labeled Aβ aggregates in both cerebral arteries and parenchymal neuritic plaques (arrowheads and arrows in Figure 4B, respectively). Higher magnification images revealed that both resorufin and methoxy-X04 were colocalized to the vascular smooth muscle cell layer, forming circumferential bands around the vessel (Figure 4D). In contrast, no staining was seen following superfusion of resorufin or methoxy-X04 in age-matched wild-type mice or young Tg2576 mice (data not shown).

### Resorufin derivatives with increased lipophilicity demonstrate improved binding affinities for CAA

**Figure 5:**
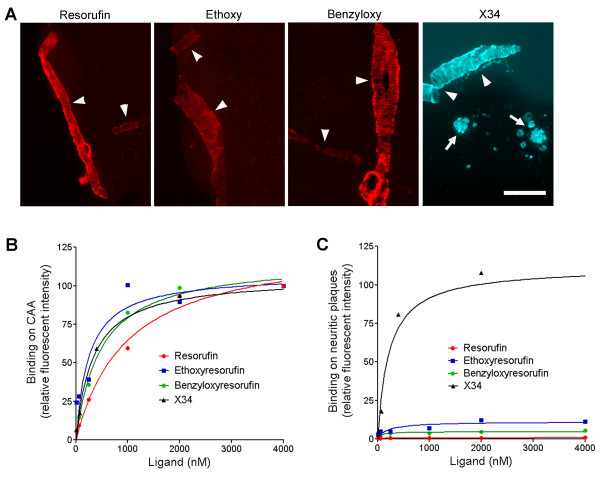
***In situ *binding assay in Tg2576 brain sections**. Sixteen month-old Tg2576 mice were perfused with PBS and brains were post-fixed in 4% paraformaldehyde. Coronal brain sections (40 μm thick) were washed three times with PBS and incubated with various concentrations of test compounds (4 sections/concentration; 6 concentrations/compound) at room temperature for 30 min. Brain sections were washed twice with PBS and 50% ethanol in PBS then cover-slipped. A. Representative images of fluorescent labeling with resorufin, ethoxy-resorufin (Ethoxy), benzyloxy-resorufin (Benzyloxy), and methoxy-X34 (X34) are seen. Note that all resorufin derivatives selectively bind CAA-laden arterioles (arrowheads), whereas the methoxy-X34 labels both CAA and neuritic plaques (arrows). Scale bar: 100 μm. Fluorescent images of the sensorimotor cortex were taken and the fluorescent intensity on CAA (B) and neuritic plaques (C) was quantified. Saturation binding curves were plotted to obtain the binding affinity (K_D_) values (see Table 2).

Several authors have postulated that moderate lipophilicity (logP_oct _in range of 1-3) is an essential characteristic of amyloid imaging tracers in order to ensure high initial brain uptake and rapid clearance from the normal brain [22,36]. To determine logP_oct _values of resorufin analogs, octanol-water partition coefficient was examined by fluorometric methods. We found that the acidic compound resorufin had weak lipophilicity (logP_oct _0.43), while ethoxy- and benzyloxy-resorufin showed increased lipophilicity (logP_oct _values of 1.94 and 2.21, respectively), indicating more appropriate partition coefficient feasible for amyloid imaging *in vivo *(Table 1). We then compared the binding affinities of resorufin analogs for CAA deposits versus neuritic plaques in fixed Tg2576 brain tissues utilizing their intrinsic fluorescence (Figure 5). Similar to our results with resorufin (Figure 1), both ethoxy-resorufin and benzyloxy-resorufin preferentially bound CAA-laden vessels but not neuritic plaques in aged Tg2576 mice (Figure 5A). The dissociation constants (K_D_) of resorufin and its derivatives were determined by the saturation binding assay performed at various concentrations (Table 2). The binding affinity of resorufin was calculated as 874 ± 177 nM (*n *= 3) on CAA deposits (Figure 5B), whereas for neuritic plaques it was > 10,000 nM (Figure 5C). The binding affinities of ethoxy- and benzyloxy-resorufin for CAA deposits were significantly higher than that of resorufin (ethoxy-resorufin: K_D _247 ± 135 nM; benzyloxy-resorufin: K_D _473 ± 82 nM). In contrast to resorufin analogs, methoxy-X34 bound almost equally to CAA deposits (K_D_: 325 ± 39 nM) and to neuritic plaques (K_D_: 219 ± 86 nM).

**Table 1:** Lipophilicity (logP_oct_) of resorufin analogs

Compound name	^a^logP_oct_
Resorufin	0.427 ± 0.036
Ethoxyresorufin	1.942 ± 0.137
Benzyloxyresorufin Methoxy-X34	2.206 ± 0.104 ^b^0.19

^a^Data represent mean ± S.E.M from three independent experiments.

^b^Data from Klunk et. al. [51].

**Table 2:** Amyloid binding affinities (K_D_) of resorufin analogs

Compound name	On CAA (nM)	On neuritic plaques (nM)
Resorufin	874 ± 177	> 10,000
Ethoxyresorufin	247 ± 135	> 10,000
Benzyloxyresorufin Methoxy-X34	473 ± 82 325 ± 39	> 10,000 219 ± 86

## Discussion

In the present study, we report three key findings: 1) that resorufin preferentially binds cerebrovascular Aβ deposits over neuritic plaques in aged Tg2576 mouse brains as well as in human AD brains; 2) that resorufin staining colocalizes to a congophilic dye methoxy-X34 in close proximity to dystrophic smooth muscle cells of CAA-affected vessels; and 3) that resorufin can be modified to enhance lipophilicity, while preserving marked selectivity for cerebrovascular Aβ deposits. These results indicate that the phenoxazine derivative resorufin and its derivatives are, to our knowledge, the first class of amyloid-imaging dyes that bind CAA in a highly selective manner. All previously described amyloid imaging ligands have been shown to bind CAA and neuritic plaques with similar affinity, making it very unlikely that these dyes could be used to develop PET imaging tracers appropriate for selective and definitive diagnosis of CAA in live patients. The unique selectivity of resorufin suggests that this class of dye has great potential as a CAA-specific amyloid tracer - the development of which would be a major diagnostic step forward for this frequent but often under-diagnosed condition.

Resorufin has been widely used as a fluorogenic probe to label bioactive molecules, and as an end-point product to measure hydrolytic activities of enzymes including peroxidases, cellulases, and aldehyde dehydrogenases. While examining the effect of CAA deposits on cerebrovascular oxidative stress, architecture, and function in aged Tg2576 mice, we initially observed that resorufin generated from Amplex red (a substrate for peroxidases) directly interacted with CAA independent of the status of oxidative stress in cerebral vessels. We characterized the cerebrovascular and parenchymal amyloid binding properties of resorufin and its derivatives ethoxy- and benzyloxy-resorufin. We found that the strongly fluorescent molecule resorufin preferentially bound cerebrovascular Aβ aggregates over neuritic plaques when *in situ *staining was performed. In an independent study, Lebouvier et. al. [37] have reported that resorufin binds to neuritic plaques, neurofibrillary tangles, and CAA in postmortem AD brain sections. However, the resorufin concentration used in that study was 2000-fold higher than that used herein (2 mM vs. 1 μM) [37]. Non-selective binding of resorufin to Aβ is expected at a high concentration; importantly, however, their study did not examine whether lower concentrations of resorufin detect Aβ deposits differentially based on localization in cerebral vessels versus brain parenchyma. We observed markedly preferential binding to CAA when staining is performed with low concentrations of resorufin under stringent conditions (i.e., washing with PBS then with 50% ethanol-containing PBS). This preferential binding for CAA cannot be attributed to artifact during brain tissue processing or fluorescent labeling since resorufin selectively visualized CAA deposits when directly applied onto the cortical surface of live Tg2576 mice (Figure 4).

Two critical conclusions stem from our observations. First, fluorescent imaging with resorufin can be a highly useful tool for the selective histopathological evaluation of CAA. For example, it is currently difficult to quantify CAA versus neuritic plaque load using conventional amyloid dyes (e.g., thioflavin-S or Congo red analogs) due to their near equal affinity for vascular versus parenchymal Aβ deposits. By exploiting the preferential binding properties of resorufin for cerebrovascular amyloid plaques, this process of CAA quantification can be performed easily. Second, our data strongly suggest that the selective binding properties of resorufin can be exploited to eventually produce a CAA-specific amyloid tracer.

CAA is a strong and independent risk factor for cerebral hemorrhage, ischemic stroke and dementia in AD and non-AD patient populations [12-18]. Excitingly, recent preclinical studies have identified several novel approaches that reduce or even prevent CAA formation [38-41]. These studies raise the intriguing possibility that one or more of these CAA-directed therapeutic strategies might eventually be tested in humans. Unfortunately, such trials would currently be limited by the difficultly in diagnosing CAA: definitive diagnosis requires brain biopsy (which is rarely clinically indicated), and "probable" diagnosis of CAA by the Boston Criteria can be made only in patients who have already suffered intracerebral hemorrhage. Given that these bleeds occur less frequently [19,42] and at a later stage [43] than ischemia, a trial using current diagnostics would be biased towards inclusion of later-stage CAA patients. The development of a non-invasive imaging technique for definitively diagnosing CAA would, in contrast, permit not only critical observational studies to better define the natural history of patients with CAA, but it would also greatly facilitate the organization and execution of therapeutic clinical trials that could include CAA patients who experience cerebral ischemia or dementia, not only hemorrhage.

To date, two chemically unrelated amyloid PET tracers, [^11^C]PIB and [^18^F]flobetapir, have demonstrated great promise as a tool for non-invasive amyloid imaging in patients with AD [25-32]. Importantly, however, these PET tracers are unable to discern whether the observed amyloid load represents neuritic plaques or CAA since they label both parenchymal and cerebrovascular amyloid deposits [44-46]. As such, our finding that resorufin analogs might represent a new class of PET agent for CAA-selective amyloid imaging is potentially groundbreaking. However, this must be considered in the context of well-described selection criteria for an ideal amyloid imaging PET tracer, including 1) high affinity and selectivity for target Aβ aggregates; 2) low molecular weight (< 400 g/mol); 3) moderate lipophilicity (logP_oct _in a range of 1-3); and 4) functional groups amenable to labeling with a positron-emitting radionucleotide such as ^11^C or ^18^F - resorufin does not yet fulfill all of these requirements due to its low binding affinity for CAA (K_D_: 874 nM) and low lipophilicity (logP_oct _of 0.43) [22]. Nevertheless, our pilot structure-activity relationship data show that chemical modification at resorufin's phenol group is able to improve binding affinity for CAA and increase lipophilicity while maintaining its high selectivity for cerebrovascular Aβ deposits. These results suggest that resorufin could serve as a lead compound to design chemical pools and to screen high-affinity, CAA-selective amyloid imaging dyes amenable to CAA imaging by PET.

Regarding the underlying mechanism by which resorufin analogs preferentially bind CAA over neuritic plaques, several potential explanations exist. One possibility is that resorufin binds fibrillar Aβ at different site(s) from other amyloid imaging dyes, a hypothesis that is supported by our observation that resorufin binding to CAA was not competitively inhibited by the congophilic dyes methoxy-X34 and methoxy-X04. A second possibility is that resorufin preferentially recognizes aggregations of Aβ_1-40 _(the predominant species in CAA) over Aβ_1-42 _(the predominant species in neuritic plaques). This hypothesis is supported by our observation that resorufin detects methoxy-X34-sensitive CAA from Tg2576 mice and humans (which is composed of both Aβ_1-40 _and Aβ_1-42 _[6,47]) (Figures 1 and 3), but does not detect methoxy-X34-sensitive CAA from BRI-Aβ42 transgenic mouse (which is composed almost exclusively of Aβ_1-42 _[5]) (data not shown). A third possibility is that resorufin directly interacts with molecules or proteins that are present in CAA but not in neuritic plaques. For example, heparan sulfate proteoglycans are expressed much more highly in cerebrovascular deposits as compared to neuritic plaques, both in human AD brains and in HCHWA-D mice carrying the Dutch-type amyloidosis [48,49]. Further investigation is required to elucidate the underlying mechanism by which resorufin preferentially binds CAA over neuritic plaques - the identification of which will not only shed new light on CAA pathophysiology but may also lead to novel therapeutic targets that could be exploited to help prevent CAA formation and its neurological consequences.

## Conclusions

In summary, to our knowledge, this is the first report demonstrating that resorufin has high selective affinity for cerebrovascular Aβ aggregates over neuritic plaques in fixed brain tissues and in live APP transgenic mice. Resorufin analogs modified at the 7-OH position demonstrated enhanced lipophilicity while retaining their high binding affinity for cerebrovascular Aβ aggregates. These results suggest that resorufin analogs are promising potential tracers for CAA-selective imaging, as opposed to conventional amyloid imaging ligands that non-selectively bind both CAA deposits and neuritic plaques. Further studies are warranted to determine whether positron-emitting radioligands such as [^11^C]- or [^18^F]-labeled resorufin analogs are feasible for use as CAA-selective PET or SPECT imaging tracers in experimental mouse models and in humans.

## Methods

### Animals and materials

All experimental protocols were approved by the Animal Studies Committee at Washington University. The production, genotyping, and background strain (B6/SJL) of Tg2576 mice used in this study have been described previously [33,35,50]. Tg2576 mice overexpressing human APP695 with the familial Swedish AD mutations at positions 670/671 under control of the hamster prion protein (PrP) promoter were a generous gift from Dr. K. Ashe (University of Minnesota, Minneapolis, MN). Resorufin, ethoxy-resorufin, benzyloxy-resorufin, and octanol were purchased from Sigma-Aldrich (St. Louis, MO). Amyloid imaging dyes methoxy-X04 and methoxy-X34 were synthesized as described previously [51].

### Fluorescent labeling and quantification of CAA and neuritic plaque loads in Tg2576 brain sections

Tg2576 and littermate wild-type mice at 12-16 months of age were anesthetized with isoflurane and transcardially perfused with PBS. Brains were removed, post-fixed overnight in 4% paraformaldehyde in 0.1 M phosphate buffer (pH 7.4) at 4°C, and preserved in 30% sucrose in 0.1 M phosphate buffer at 4°C. Brains were sectioned coronally (40 μm) on a freezing sliding microtome as described previously [52]. To label brain sections with resorufin and methoxy-X34, fixed brain tissues (4 sections/brain) were washed three times with PBS and permeabilized by incubating in PBS containing 0.25% Triton-X100 (PBS-T) at room temperature for 30 min. Brain sections were stained with PBS-T containing 1 μM resorufin and 2 μM methoxy-X34 at room temperature for 30 min. Brain sections were washed three times with PBS and once with 50% ethanol in PBS for 5 min each. After three more PBS washes, brain sections were mounted on a slide glass and cover-slipped with Vectashield mounting media (Vector laboratories, Burlingame, CA). Fluorescent staining was visualized using a Nikon Eclipse ME600 digital video microscopy system (Nikon Instruments Inc., Melville, NY) and MetaMorph imaging software (Molecular Devices, Sunnyvale, CA). Cross-sectional area covered by CAA vessels and parenchymal plaques were quantified using ImageJ software (National Institutes of Health, Bethesda, MD) as previously described [6].

### Triple labeling and multi-photon microscopy

Vascular smooth muscle cells and CAA deposition was assessed as previously reported [35] with modification. Paraformaldehyde-fixed whole brains were permeabilized with PBS-T for 20 min at room temperature, and then incubated with PBS-T containing 1 μM resorufin and 2 μM methoxy-X34 for 30 min at room temperature. Brains were washed three times with PBS and once with 50% ethanol in PBS for 5 min each. Brains were blocked with 2% bovine serum albumin (BSA) in PBS-T for 30 min, followed by incubation with phalloidin-Alexa-488 (Invitrogen, Carlsbad, CA) in PBS containing 1% BSA. Fluorescent staining with phalloidin-Alexa488, resorufin and methoxy-X34 was simultaneously imaged using a Zeiss LSM 510 META LNO two-photon microscope (Carl Zeiss, Jena, Germany).

### Immunofluorescent labeling in human AD brain sections

Paraffin-embedded postmortem brain sections prepared from patients with AD were provided by the Alzheimer's Disease Research Center at Washington University. Brain tissues (10 μm thick) were deparaffinized with xylene and rehydrated by incubation with 100-70% ethanol and PBS. Brain sections were blocked with buffer (PBS-BB) containing 0.1% Triton-X 100, 0.2% dry milk and 1% BSA serum in PBS at room temperature for 1 h. To label fibrillar amyloid, sections were then incubated with biotinylated anti-Aβ antibody 3D6 (dilution: 1:3000, a generous gift from Dr. David M. Holtzman) overnight at 4°C. After washing with PBS, sections were incubated with streptavidin-Alexa 488 (Invitrogen, Carlsbad, CA), followed by labeling with 1 μM resorufin as described above. Sections were rinsed with PBS, cover-slipped and subjected to fluorescent microscopy.

### Closed cranial window preparation and live microscopic imaging

A closed cranial window preparation was performed as previously reported [35]. Briefly, mice were anesthetized with isoflurane (4% induction, 1.5% maintenance), and a 4-mm diameter craniotomy was performed with a water-cooled dental drill in the right parietal bone. Two silastic tubings (ID: 0.3 mm, OD: 0.64 mm; Dow Corning, Midland, MI) were inserted through the bone wax to permit topical application of vasodilators. The craniotomy was filled with artificial cerebrospinal fluid (aCSF) and sealed to the bone with a microscope coverglass using dental cement. To label amyloid deposits in the brain, mice were administered BBB-permeable Congo red derivative methoxy-X04 (6 mg/kg i.p.) as described [35]. Fifteen hours later, mice were re-anesthetized with isoflurane and α-chloralose, and ventilated. Fluorescent images were visualized using a Nikon Eclipse 600 ME digital video microscopy system. To label CAA, aCSF containing 1 μM resorufin was infused into the cranial window at a rate of 20 μl/min for 5 min. After washing with aCSF for 10 min, fluorescent resorufin and methoxy-X04 images were taken.

### Determination of lipophilicity (logP_oct_)

Octanol-water partition coefficients (logP_oct_) were determined as described previously with modification [53]. An equal volume (600 μl) of *n*-octanol (Sigma-Aldrich, St. Louis, MO) and distilled water were added to a microcentrifuge tube, followed by an addition of resorufin analogues at a final concentration of 100 μM. The samples were incubated at room temperature with a brief vortex every 5 min for 60 minutes. After centrifugation at 2,000 *g *for 10 min, octanol and water layers were separately transferred to microcentrifuge tubes. Concentrations were determined by fluorometry at an excitation wave length of 530/25 nm and an emission wave length of 590/30 nm using an ELISA reader (Biotek, Winooski, VT).

## List of abbreviations used

AD: Alzheimer's disease; CAA: cerebral amyloid angiopathy; PET: positron emission tomography; VSMC: vascular smooth muscle cell; PBS: phosphate-buffered saline; PBS-T: PBS containing 0.25% Triton-X100; aCSF: artificial cerebrospinal fluid.

## Competing interests

BHH, WC, RHM and GJZ have patent applications on the composition, methods, and use related to resorufin derivatives. The authors declare that they have no competing interests.

## Authors' contributions

BHH contributed to the general administration and direction of the project, interpretation of experimental results, and development and writing of the manuscript. MLZ performed live fluorescent imaging. AKV performed multi-photon imaging in fixed brain sections and image processing, and writing of the manuscript. EM contributed to fluorescent imaging, statistical data analysis, and writing the manuscript. DHK performed the fluorescent ligand binding assays. JKG performed the CAA load and neuritic plaque load analyses. WC contributed to the experimental design for structure-activity relationship of test compounds. RHM contributed to the overall design of chemical modification and review of data. GJZ contributed to direction of the project, interpretation of experimental results, and critical reviewing of the manuscript. All authors read and approved the final manuscript.
